# Relationship Between Platelet Count and In-hospital Mortality in Adult Patients With COVID-19: A Retrospective Cohort Study

**DOI:** 10.3389/fmed.2022.802412

**Published:** 2022-06-14

**Authors:** Qilin Yang, Jun Gao, Xiaomei Zeng, Junyu Chen, Deliang Wen

**Affiliations:** ^1^Department of Critical Care, The Second Affiliated Hospital of Guangzhou Medical University, Guangzhou, China; ^2^Department of Nephrology, Peking University International Hospital, Beijing, China

**Keywords:** platelet count, in-hospital mortality, ferritin, coronavirus disease 2019, systemic inflammation

## Abstract

**Background:**

The coronavirus disease 2019 (COVID-19) has become a global pandemic. Systemic inflammation in COVID-19 patients has been associated with poor clinical outcome. This study aims to determine the relationship between platelet count and in-hospital mortality.

**Methods:**

The original data of this study were from article development and validation of a predictive model of in-hospital mortality in COVID-19 patients. In this secondary analysis, we adopted multi-variable logistic regression analyses and smooth curve fitting to assess the independent association between platelet count and in-hospital mortality. We further applied a two-piecewise linear regression model to examine the nonlinear association between platelet count and in-hospital mortality.

**Results:**

Of the 2006 patients, the average age of the participants was 65.9 ± 16.5 years and 42.6% were women. We observed a U-shaped relationship between platelet count and in-hospital mortality. We found two different slopes, the correlations between platelet count and in-hospital mortality of COVID-19 patients were totally different below and above the inflection point which was around 370 × 10^9^/L. On the left side of the inflection point, the OR was 0.996 (OR: 0.996, 95%CI: 0.994–0.998, *p* < 0.001). On the right side of the inflection point, the OR was 1.011 (OR: 1.011, 95%CI: 1.001–1.021, *p* = 0.029).

**Conclusions:**

A U-shaped association between platelet count and in-hospital mortality was found in the patients with COVID-19. The optimal of platelet count associated with the lowest risk of in-hospital mortality was around 370 × 10^9^/L.

## Background

The coronavirus disease 2019 (COVID-19), an infectious disease caused by a novel strain of human coronavirus, has become the focus of attention worldwide (1). Systemic inflammation in COVID-19 patients has been associated with poor clinical outcome (2–4). Platelets, nucleate megakaryocyte fragments circulating in the blood, play a crucial role in inflammatory diseases (5). There is growing recognition of the critical role of platelets in inflammation and immune responses (6). Previous studies have shown that platelet count is correlated with COVID-19 mortality (7–9).

In the general (10), COPD (11), venous thromboembolism (12), and elderly (13) populations, a U-shaped association was recognized between platelet count and mortality, though its role in COVID-19 remains unclear. Using data from the study of development and validation of a predictive model of in-hospital mortality in COVID-19 patients (14), a respective cohort study enriched for the presence of comorbidity and containing adjudicated events, we investigated *post-hoc* the association of platelet count measured at basement with in-hospital mortality.

## Participants and Methods

### Data Source

The original data of this study were from the development and validation of a predictive model of in-hospital mortality in COVID-19 patients study (14). Since Diego et al. have relinquished the ownership of the original dataset to PLoS ONE (https://journals.plos.org/plosone/s/data-availability), we can use this dataset to perform secondary analysis based on different scientific hypotheses. The original study was granted an exempt status and the requirement for obtaining informed consent was waived by the Ethics Committee for Clinical Research of the Hospital Universitario Fundación Jiménez Díaz (14). We followed the Strengthening the Reporting of Observational Studies in Epidemiology guidelines to report this study (15).

### Study Population

The original study retrospectively evaluated consecutive hospitalized patients with confirmed moderate or severe COVID-19 from four hospitals [Hospital General de Villalba (Collado Villalba, Madrid), Hospital Infanta Elena (Valdemoro, Madrid), Hospital Universitario Rey Juan Carlos (Móstoles, Madrid), and Hospital Universitario Fundación Jiménez Díaz in Madrid] from 27 February to 17 April 2020. The diagnosis of COVID-19 was based on World Health Organization interim guidance and confirmed by RNA detection of 2019-nCoV in the clinical laboratory of Hospital Universitario Fundación Jiménez Díaz. Diego et al. extracted de-identified data from the Huawei (Huawei Technologies Co., Ltd., Shenzhen, China) platform and the collaboration of Indizen-Scalian (Madrid, Spain). Four patients younger than 18 years old and 60 patients missing platelet count data were excluded in further analysis.

### Variable Extraction

#### Baseline Platelet Count

Baseline platelet count was first collected either in the emergency department or within 3 days from admission to a ward from electronic medical records (14).

### Covariates

We included the following variables based on published literature and clinical experience: demographic characteristics and chronic comorbidities (arterial hypertension, diabetes mellitus, smoking habit, cardiovascular disease, and pulmonary disease). Laboratory values included body mass index (BMI), lactate dehydrogenase (LDH), ferritin, D-dimer, absolute lymphocyte count, estimated glomerular filtration rate (eGFR), activated partial thromboplastin time (APTT), and fibrinogen.

### Outcome

The outcome was in-hospital mortality which was monitored up to 17 April 2020 (14).

### Statistical Analysis

Descriptive analysis was performed for all patients. Categorical variables were expressed as numbers and percentages. Continuous variables were expressed as mean and standard deviation (SD) for normal distributions or median and interquartile range for skewed distributions. We used the chi-square test, one-way ANOVA, and Kruskal–Wallis test for the comparison of categorical, normally distributed, and non-normally distributed continuous variables, respectively. We used dummy variables to indicate missing covariate values (16).

We adopted multi-variable logistic regression analyses and smooth curve fitting to assess the independent association between platelet count and in-hospital mortality. We further applied a two-piecewise linear regression model using a smoothing curve to examine the nonlinear association between platelet count and in-hospital mortality. A likelihood ratio test was conducted to compare the one-line linear regression model with the two-piecewise linear model. All the analyses were performed with the statistical software packages R 3.3.2 (http://www.R-project.org, The R Foundation) and Free Statistics software version 1.3 (17). A two-tailed test was performed and *p* < 0.05 was considered statistically significant.

## Results

### Baseline Characteristics of Participants

From the original cohort, after excluding 4 patients younger than 18 years old and 60 patients missing platelet count data on admission, 2,006 patients were included in our study. Among all these patients, the average age of the participants was 65.9 ± 16.5 years and 42.6% were women. A total of 3.7% of patients were smokers. Compared with the first platelet count group, the fourth platelet count group contained more women, cardiovascular disease, higher D-dimer, EGFR, lymphocyte, and LDH levels, and less pulmonary disease.

### Outcome

**Figure 1:**
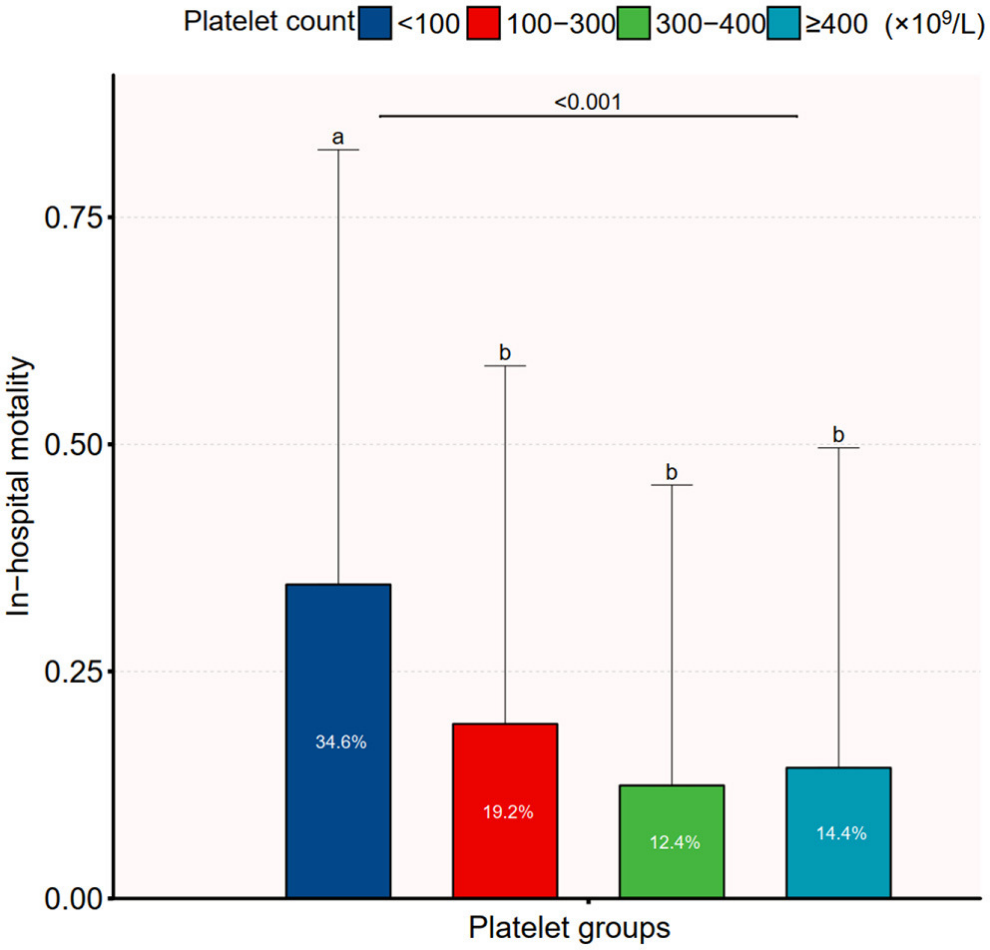
In-hospital mortality in different platelet count groups.

The overall in-hospital mortality was 18.7%. Figure 1 shows the in-hospital mortality in different platelet count groups. The in-hospital mortality in groups 1–4 was 34.6, 19.2, 12.4, and 14.4%, respectively. The results of the univariate and multivariate logistic regression models are shown in Table 1 and **Table 4**. In the fully adjusted model (adjusted for all covariates in Table 2), categorized platelet count in the multivariate logistic regression model seemed to confirm a non-linear relationship between platelet count and in-hospital mortality. The 300–400(× 10^9^/L) platelet count group had the lowest in-hospital mortality.

**Table 1:** Multivariable logistic regression models evaluating the association between platelet count and in-hospital mortality.

**Variable**	**Total** ***n***	**In-hospital mortality *n* (%)**	**Model I** **OR (95%CI)**	***P*** **-value**	**Model II** **OR (95%CI)**	***P*** **-value**	**Model III** **OR (95%CI)**	***P*** **-value**
Platelet <100 (× 10^9^/L)	81	28 (34.6)	3.72 (2.03–6.81)	<0.001	3.03 (1.54–5.98)	0.001	3.65 (1.74–7.66)	0.001
Platelet 100–300 (× 10^9^/L)	1561	300 (19.2)	1.67 (1.10–2.54)	0.015	1.58 (1.00–2.48)	0.048	1.93 (1.18–3.16)	0.009
Platelet 300–400 (× 10^9^/L)	225	28 (12.4)	1.00 (Ref)		1.00 (Ref)		1.00 (Ref)	
Platelet ≥400 (× 10^9^/L)	139	20 (14.4)	1.18 (0.64–2.19)	0.595	1.19 (0.61–2.33)	0.613	1.50 (0.73–3.09)	0.270

*Model I, No adjustment*.

*Model II, Adjusted for age and sex*.

*Model III, Adjusted for all covariates in Table 2*.

**Table 2:** Baseline characteristics of platelet count analysis.

**Variables**		**Baseline platelet count (× 10** ^**9**^ **/L)**				
	**Total**	**<100**	**100–300**	**300–400**	**≥400**	
	**(*n* = 2,006)**	**1 (*n* = 81)**	**2 (*n* = 1,561)**	**3 (*n* = 225)**	**4 (*n* = 139)**	***P*** **-value**
Age (years)	65.9 ± 16.5	69.3 ± 17.0	66.0 ± 16.5	64.5 ± 16.8	64.4 ± 16.6	0.108
Sex (Female), *n* (%)	854 (42.6)	27 (33.3)	655 (42.0)	113 (50.2)	59 (42.4)	0.038
Smoking, *n* (%)	78 (3.9)	3 (3.7)	62 (4.0)	9 (4.0)	4 (2.9)	0.964
Cardiovascular disease, *n* (%)	312 (15.6)	20 (24.7)	234 (15)	39 (17.3)	19 (13.7)	0.097
Pulmonary disease, *n* (%)	332 (16.6)	17 (21)	271 (17.4)	32 (14.2)	12 (8.6)	0.027
Diabetes, *n* (%)	400 (19.9)	17 (21)	298 (19.2)	50 (22.2)	35 (25.2)	0.287
Hypertension, *n* (%)	895 (44.6)	40 (49.4)	695 (44.5)	98 (43.6)	62 (44.6)	0.840
BMI (Kg/m^2^)	28.2 ± 6.0	28.5 ± 5.2	28.3 ± 6.0	28.6 ± 6.8	26.6 ± 4.7	0.157
D-dimer (μg/l)	623 (336, 1,106)	807 (424, 1,442)	567.0 (318, 1,016)	730 (391, 1,330)	1002 (593, 2,110)	<0.001
Ferritin (ng/ml)	775 (394, 1,484)	879 (425, 1,603)	775.0(376, 1,476)	756 (388, 1,428)	783 (532, 1,430)	0.597
EGFR (ml/min/l.73m^2^)	89.7(76.4, 101.2)	89.7 (76.4, 100.3)	88.7 (75.4, 99.9)	94.4 (80.4, 103.6)	94.7 (78.9, 105.3)	0.002
Lymphocyte (× 10^9^/L)	1.0 (0.7, 1.3)	0.7 (0.5, 1.1)	0.9 (0.7, 1.3)	1.1 (0.8, 1.4)	1.1 (0.7, 1.5)	<0.001
LDH (U/L)	298 (233, 384)	278 (209, 352)	293 (232, 380)	320 (257, 409)	326 (248, 399)	<0.001
Fibrinogen (mg/dl)	703.5 ± 206.0	615.1 ± 167.4	690.3 ± 191.7	775.6 ± 253.7	786.6 ± 241.6	<0.001
APTT (seconds)	31.1 ± 7.9	32.5 ± 5.9	31.2 ± 8.6	30.3 ± 4.4	30.7 ± 4.7	0.158

*BMI, body mass index; eGFR, estimated glomerular filtration rate; LDH, lactate dehydrogenase; APTT, activated partial thromboplastin time; SD, standard deviation*.

We tried to look at different thresholds to identify patients at risk and used <100 × 10^9^ /L vs. 100–550 × 10^9^/L vs. >550 × 10^9^/L for sensitivity analysis. Compared with 100–550 × 10^9^ /L groups, the ORs of <100 × 10^9^/L and >550 × 10^9^ / L were 2.34 (1.35–4.07) and 1.69 (0.51–5.6) after adjusting for all covariates in Table 2.

### The Nonlinearity Relationship Between Platelet Count and In-hospital Mortality

**Figure 2:**
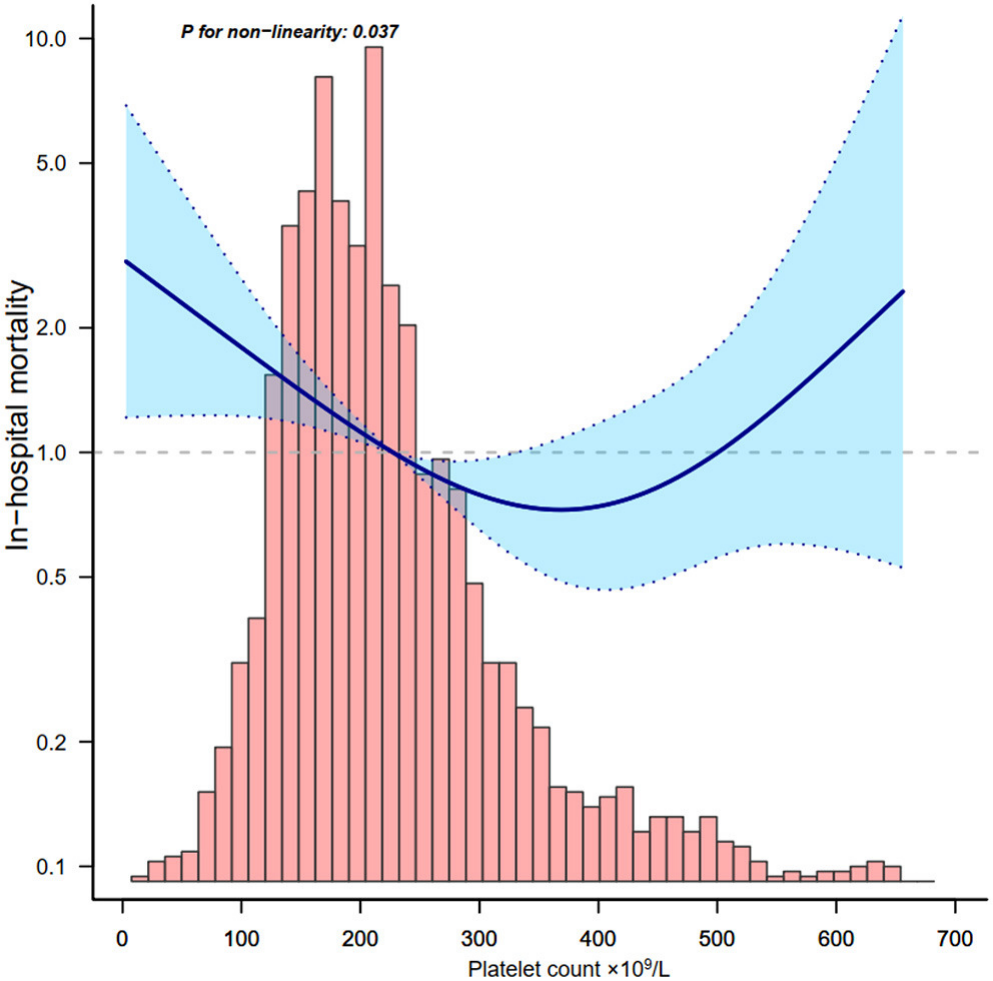
Relationship between platelet count and in-hospital mortality. Adjusted for all covariates in Table 2.

Through the multivariate logistic regression model and smooth curve fitting, we observed that the relationship between platelet count and in-hospital mortality was non-linear (Figure 2). Data were fit to a piecewise multivariate logistic regression model and found two different slopes. In our study, the *P*-value for the non-linear test was 0.037 (Table 3), we thus used a two-piecewise model to fit the link between platelet count and in-hospital mortality. We found an inflection point at about 370 × 10^9^/L (Figure 2). On the left side of the inflection point, the OR was 0.996 (OR: 0.996, 95%CI: 0.994–0.998, *p* < 0.001). On the right side of the inflection point, the OR was 1.011 (OR: 1.011, 95%CI: 1.001–1.021, *p* = 0.029). It suggests that the risk of in-hospital mortality started to decrease by 0.4% per 1 × 10^9^/L platelet change until a platelet count of ~370 × 10^9^/L. Then the risk of in-hospital mortality started to increase by 1.1% per 1 × 10^9^/L platelet change (*P*-value for non-linear test was 0.037).

**Table 3:** The non-linearity relationship between platelet count and in-hospital mortality.

**Threshold of driving pressure**	**OR**	**95% CI**	***P*** **-value**
<370 × 10^9^/L	0.996	0.994–0.998	<0.001
≥370 × 10^9^/L	1.011	1.001–1.021	0.029
Non-linear test			0.037

*Adjusted for all covariates in Table 2*.

### Other Risk Factors for In-hospital Mortality in Patients With COVID-19

Univariate logistic and multivariable logistic regression analysis of risk factors for in-hospital mortality in patients with COVID-19 is reported in Table 4. We found age, male, history of pulmonary disease, history of diabetes, D-dimer, and LDH were independent risk factors for in-hospital mortality in this cohort (all *P* < 0.05).

**Table 4:** Univariate logistic and multivariable logistic regression models evaluating the association between platelet count and in-hospital mortality.

**Variable**	**Univariate logistic analysis**	***P*** **-value**	**Multivariable logistic analysis**	***P*** **-value**
	**OR (95%CI)**		**OR (95%CI)**	
Platelet <100 (× 10^9^/L)	3.72 (2.03–6.81)	<0.001	3.65 (1.74–7.66)	0.001
Platelet 100–300 (× 10^9^/L)	1.67 (1.10–2.54)	0.015	1.93 (1.18–3.16)	0.009
Platelet 300–400 (× 10^9^/L)	1.00 (Ref)		1.00 (Ref)	
Platelet ≥400 (× 10^9^/L)	1.18 (0.64–2.19)	0.595	1.50 (0.73–3.09)	0.27
Age (years)	1.08 (1.07–1.09)	<0.001	1.09 (1.07–1.101)	<0.001
Sex (Female), *n* (%)	0.74 (0.59–0.92)	0.008	0.53 (0.39–0.70)	<0.001
Smoking, *n* (%)	1.07 (0.62–1.84)	0.8054	1.16 (0.58–2.31)	0.682
Cardiovascular disease, *n* (%)	2.30 (1.76–3.00)	<0.001	0.87 (0.62–1.22)	0.407
Pulmonary disease, *n* (%)	1.36 (1.03–1.80)	0.0311	1.43 (1.01–2.01)	0.042
Diabetes, *n* (%)	2.08 (1.62–2.67)	<0.001	1.39 (1.02–1.90)	0.040
Hypertension, *n* (%)	2.83 (2.25–3.56)	<0.001	0.97 (0.71–1.32)	0.830
BMI (Kg/m^2^)	0.99 (0.98–1.00)	0.022	0.997 (0.96–1.03)	0.845
D-dimer (μg/l)	1.002 (1.001–1.003)	<0.001	1.003 (1.002–1.004)	<0.001
Ferritin (ng/ml)	1.00 (0.9999–1.0001)	0.705	1.00(0.9999–1.0002)	0.459
EGFR (ml/min/l.73m^2^)	0.98 (0.978–0.983)	<0.001	0.998 (0.989–1.01)	0.721
Lymphocyte (× 10^9^/L)	1.04 (0.98–1.10)	0.234	1.06 (0.98–1.15)	0.181
LDH (U/L)	1.002 (1.001–1.003)	<0.001	1.004 (1.003–1.004)	<0.001
Fibrinogen (mg/dl)	1.00(0.999–1.000)	0.044	1.00(0.999–1.001)	0.600
APTT (seconds)	0.997 (0.988–1.007)	0.613	1.009 (0.995–1.024)	0.193

**Figure 3:**
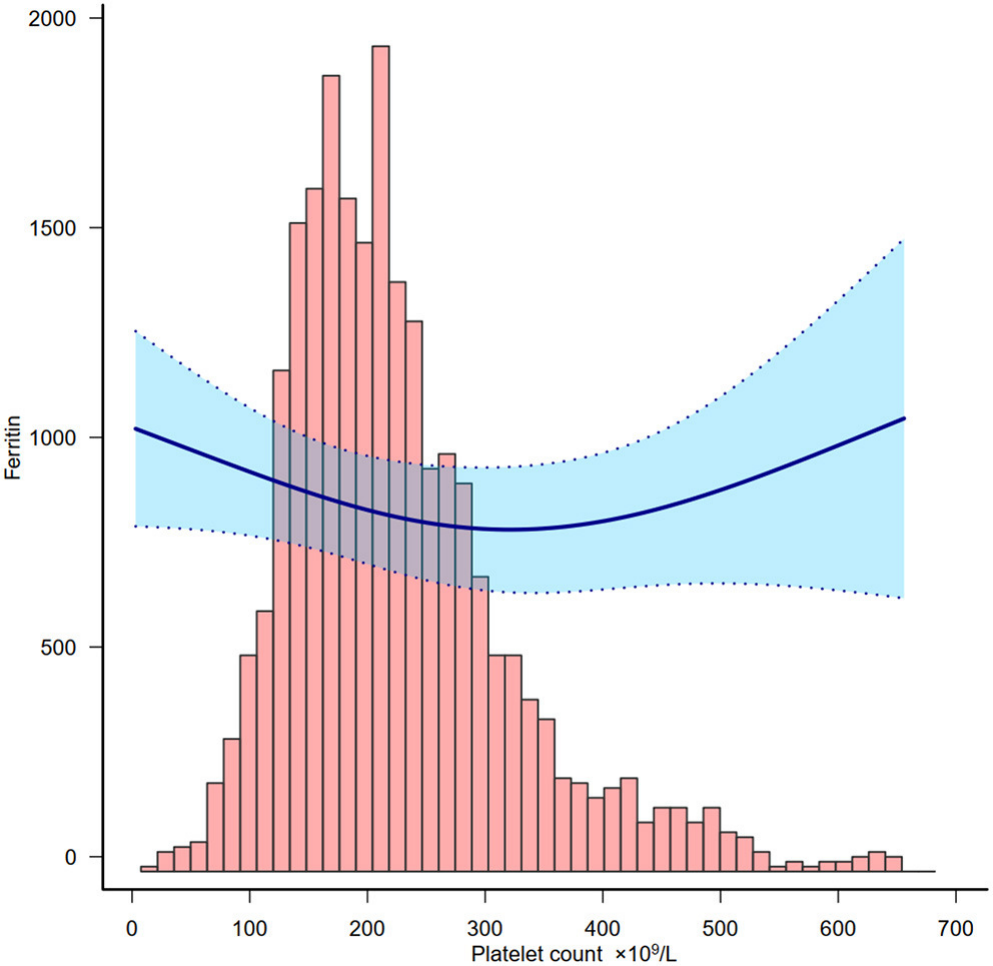
Relationship between platelet count and ferritin. Adjusted for all covariates in Table 2 except ferritin.

We also detected the association between platelet count and ferritin in order to understand the relationship between platelet count and inflammation. Based on Figure 3, platelet count was negatively associated with ferritin below 300 × 10^9^/L.

## Discussion

In this observational retrospective cohort study, we tried to examine the optimal platelet count associated with in-hospital mortality in patients with COVID-19. A U-shaped association between platelet count and in-hospital mortality was found in the cohort. The correlations between platelet count and in-hospital mortality of COVID-19 patients were totally different below and above the inflection point which was around 370 × 10^9^/L. Platelet count, as assessed at baseline, was negatively associated with in-hospital mortality of COVID-19 patients below 370 × 10^9^/L, and it was positively associated above 370 × 10^9^/L. The optimal platelet count associated with the lowest risk of in-hospital mortality was around 370 × 10^9^/L.

Low platelet count is a common laboratory finding in patients with severe COVID-19 (18). Our study found that 4% (81) of patients had a platelet count <100 × 10^9^/L. In a previous study, thrombocytopenia was associated with poor outcome in patients with COVID-19 (18–21). Their results are akin to part of our findings where platelet counts <100 × 10^9^/L correlated with the highest in-hospital mortality. However, most of the studies converted platelet count into dichotomous variables and only compared the outcome of platelet counts <100 × 10^9^/L (22) or 125 × 10^9^/L (23) or 150 × 10^9^/L (24) with other COVID-19 patients. It was hard in these studies to find either a non-linearity relationship between platelet count and in-hospital mortality or an optimal platelet count associated with the lowest risk of in-hospital mortality.

We used smooth curve fitting (25) and a two-piecewise linear regression model (25) to determine that a high platelet count may also lead to increased mortality in patients with COVID-19. Whereas, in multivariable logistic regression analysis, we only found an increasing trend of mortality in group 4 (platelet ≥400 × 10^9^/L) (OR: 1.5, 95%CI: 0.73–3.09, *P* = 0.27). This instability may be the result of the highly sensitive smooth curve fitting in analyzing change trends.

In fact, a U-shaped association between platelet count and in-hospital mortality is universal in different diseases. Several studies have found a non-linear relationship between platelet count and outcomes. Fawzy et al. (11) demonstrated a U-shaped association with platelet count and risk of 3-year all-cause mortality in stable COPD. Van et al. found low and high platelet counts were associated with non-cardiovascular mortality in the elderly, including cancer mortality. Di Micco et al. (12) found a U-shaped relationship between platelet count and the 3-month rate of major bleeding and fatal bleeding in patients with VTE.

The association between platelet count and clinical outcome may not reveal any causality as many parameters could both be the cause and/or the consequence of the changing platelet count. For example, thrombocytopenia could be due to an inflammatory response. The pathophysiology of thrombocytopenia in COVID-19 is hypothetically caused by the alteration of platelet production and consumption (and/or destruction) (26). It affects platelet production by either directly or indirectly affecting hematopoietic stem cells (HSCs), reducing thrombopoietin production, and megakaryocyte maturation due to an increase of specific inflammatory cytokines (27). In this study we also explored the relationship between platelet count and ferritin which is a bio-marker of inflammation and found that a lower platelet count represented higher inflammation in this cohort.

Similar to other studies, we found age (21), male gender (28), and underlying chronically illness (29) were risk factors of in-hospital mortality of patients with COVID-19. Our study also found that D-dimer, representing thrombotic risk (30), and LDH, representing systemic inflammation (31), were also associated with prognosis in multivariable logistic regression analysis. These results suggest that age, sex, underlying chronically illness, D-dimer, and LDH deserve further study.

Our research has the following limitations that need attention. First, residual confounders potentially exist, as with all retrospective analyses. By maximizing the sample size, we adjusted for all possible confounders we could. Second, our data are only from Spain and cannot cover other populations. Third, the association between platelet count and clinical outcome may not reveal any causality. However, we tried various techniques such as the non-linearity relationship test and used different thresholds group analyses to confirm this relationship which is worthy of further investigation.

## Conclusion

A U-shaped association between platelet count and in-hospital mortality was found in patients with COVID-19. The optimal platelet count associated with the lowest risk of in-hospital mortality was around 370 × 10^9^/L.

## Data Availability Statement

The original contributions presented in the study are included in the article/supplementary material, further inquiries can be directed to the corresponding author/s.

## Ethics Statement

Ethical review and approval was not required for the study on human participants in accordance with the local legislation and institutional requirements. Written informed consent from the patients/participants or their legal guardian/next of kin was not required to participate in this study in accordance with the national legislation and the institutional requirements.

## Author Contributions

QY designed the study and wrote the manuscript. JG conducted data analysis and wrote the manuscript. XZ drew the figures. JC conducted data analysis. DW conducted data interpretation and modified the manuscript. All authors contributed to the article and approved the submitted version.

## Conflict of Interest

The authors declare that the research was conducted in the absence of any commercial or financial relationships that could be construed as a potential conflict of interest.

## Publisher's Note

All claims expressed in this article are solely those of the authors and do not necessarily represent those of their affiliated organizations, or those of the publisher, the editors and the reviewers. Any product that may be evaluated in this article, or claim that may be made by its manufacturer, is not guaranteed or endorsed by the publisher.

## Abbreviations

COVID-19: the coronavirus disease 2019
COPD: chronic obstructive pulmonary disease
VTE: venous thromboembolism
BMI: body mass index
LDH: lactate dehydrogenase
eGFR: estimated glomerular filtration rate
APTT: activated partial thromboplastin time
SD: standard deviation
HSCs: hematopoietic stem cells.
